# 3D Reconstruction of Slug Flow in Mini-Channels with a Simple and Low-Cost Optical Sensor

**DOI:** 10.3390/s19204573

**Published:** 2019-10-21

**Authors:** Huajun Li, Yandan Jiang, Haifeng Ji, Guangyu Liu, Shanen Yu

**Affiliations:** 1 School of Automation, Hangzhou Dianzi University, Hangzhou 310018, China; g.liu@hdu.edu.cn (G.L.); shanen_yu@hdu.edu.cn (S.Y.); 2 College of Control Science and Engineering, Zhejiang University, Hangzhou 310027, China; ydjiang@zju.edu.cn (Y.J.); hfji@zju.edu.cn (H.J.)

**Keywords:** gas-liquid two-phase flow, mini-channels, optical sensor, 3D image reconstruction, slug flow, Support Vector Machine

## Abstract

The present work provides a new approach for 3D image reconstruction of gas-liquid two-phase flow (GLF) in mini-channels based on a new optical sensor. The sensor consists of a vertical and a horizontal photodiode array. Firstly, with the optical signals obtained by the vertical array, a measurement model developed by Support Vector Regression (SVR) was used to determine the cross-sectional information. The determined information was further used to reconstruct cross-sectional 2D images. Then, the gas velocity was calculated according to the signals obtained by the horizontal array, and the spatial interval of the 2D images was determined. Finally, 3D images were reconstructed by piling up the 2D images. In this work, the cross-sectional gas-liquid interface was considered as circular, and high-speed visualization was utilized to provide the reference values. The image deformation caused by channel wall was also considered. Experiments of slug flow in a channel with an inner diameter of 4.0 mm were carried out. The results verify the feasibility of the proposed 3D reconstruction method. The proposed method has the advantages of simple construct, low cost, and easily multipliable. The reconstructed 3D images can provide detailed and undistorted information of flow structure, which could further improve the measurement accuracy of other important parameters of gas-liquid two-phase flow, such as void fraction, pressure drop, and flow pattern.

## 1. Introduction

Gas-liquid two-phase flow (GLF) in micro-channels and mini-channels has been getting more attention recently due to the rapid development of miniature equipment applied in medical devices, heat exchangers, cooling systems, and chemical reactors, etc. [1,2]. In the past few decades, researchers have proved that miniature equipment with integrated channels intensifies heat and mass transfer, and, hence, has better process performance. Due to the predominance of surface tension over gravity force, the flow characteristics of GLF in micro-/mini-channels differ from those of the conventional channels. A better understanding of GLF in micro-/mini-channels is essential for flow mechanism research, process control, and monitoring, which depends on accurate and real-time measurement methods [3,4]. Therefore, there is an urgent need for developing effective measurement methods, which should be low-cost, fast, easily multipliable, and suitable for miniature equipment.

Flow visualization is an effective approach for having a direct understanding of the flow, which can also provide detailed information about flow structure, flow pattern, and phase interface, etc. In conventional channels, many visualization or tomography methods, such as high-speed photography, optical tomography, electrical resistance tomography, and wire-mesh sensors, have been applied in diverse industrial fields [5]. High-speed visualization utilizes a high-speed camera and a backlighting arrangement to record images of two-phase flow. Based on the assumption of bubbles shaped as a spheroid, the images are further processed to build flow structure. To consider the gravity effect on phase boundary, Fu et al. used a high-speed camera and an isosceles right-angle prism to record two views of two-phase flow simultaneously [6]. Then they processed the images from the two views and reconstructed 3D images of the bubble flow. The application of the high-speed camera is limited by its expensive cost and complex image processing, which is usually a laboratory apparatus. Optical tomography is based on the radiation-attenuation principle, and it is one of the “hard-field” modalities, which refers to the condition where the sensitivity field is independent of the distribution of the measured components [5]. Ruzairi et al. presented an optical tomography sensor, which consisted of two orthogonal and two rectilinear optical arrays. They further used a hybrid image reconstruction algorithm to reconstruct 2D images [7]. For better resolution, Schleicher et al. proposed a fast optical tomography sensor with 256 light emitters and 32 light receivers. They reconstructed 2D images (slices) firstly and then piled up the 2D images to reconstruct 3D images [8]. However, the flow velocity was not considered, and their 3D images are virtually not spatial 3D images. Electrical tomography reconstructs images based on the different electrical characteristics of the medium and can be categorized as “soft-field” tomography, which means that the sensitivity of the medium imaged depends on the distribution of the measured components in the process being imaged [5]. The variants of electrical tomography include electrical capacitance tomography (ECT), electrical resistance tomography (ERT), and electrical impedance tomography (EIT) [9]. In particular, Banasiak et al. proposed a flow visualization and identification method based on 3D electrical capacitance tomography. They reconstructed the 3D ECT volumetric images with nonlinear electrical capacitance tomography reconstruction algorithms and further compared their results with images from a CCD camera [10]. The aforementioned methods obtained satisfactory performance in conventional channels and have been widely applied in many industrial fields. The electrical tomography methods encounter great challenges and difficulties in micro/mini channels due to the small interspace of miniature devices. In micro/mini channels, the number and size of the electrodes will be limited, which leads to weak signals and low sensitivity. In conclusion, the conventional visualization methods are either expensive or difficult to be applied in micro/mini channels. It is necessary to propose a low cost and effective 3D reconstruction method for GLF in micro/mini channels to meet the increasing requirements of academic research and industrial applications.

Among the aforementioned visualization methods, optical tomography methods occupy the advantages of low-cost, high sample frequency, and high spatial resolution. Especially with the fast development of semiconductor technology and elaborate processing, integrated and miniature optical sensors provide a promising approach for 2D/3D reconstruction in restricted space. However, corresponding research is insufficient, and most of the reported optical methods focus on conventional channels [7,9,11,12]. The present work presents a new approach for 3D visualization of GLF in mini-channels based on a new optical sensor with a horizontal and vertical photodiode array. The proposed 3D visualization method utilizes the vertical array to acquire the information of the cross-sectional interface and then builds 2D images of the GLF. The interval between 2D images is determined by the horizontal array. Finally, 3D images are reconstructed by piling up the 2D images. The proposed method provides detailed and undistorted 3D images of GLF in mini-channels, which can further improve the measurement performance of other important parameters, such as void fraction, flow pattern, and phase distribution. The proposed method has the advantages of low cost, fast, and easy multipliable.

## 2. Experimental Apparatus and Optical Measurement System

### 2.1. Experimental Apparatus

**Figure 1:**
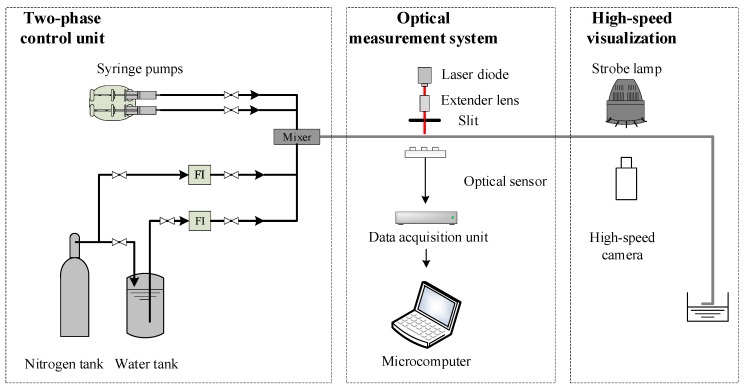
Experimental apparatus of the gas-liquid two-phase flow.

Figure 1 illustrates the experimental apparatus developed in this work, which consists of a two-phase flow control unit, an optical measurement system and a high-speed visualization part. The control unit has a syringe pump, a nitrogen/water tank, two flowmeters, and a mixer. To cover a wide range of two-phase flow rate, the setup occupies two optional methods for driving the flow through the test channel horizontally: (i) using speed-controlled syringe pumps under low flowrate conditions, and (ii) using the nitrogen tank with relatively stable pressure under high flowrate conditions. Nitrogen and water were chosen as the test materials. The experiments were carried out at a temperature of 25 °C and atmospheric pressure.

### 2.2. Optical Measurement System

**Figure 2:**
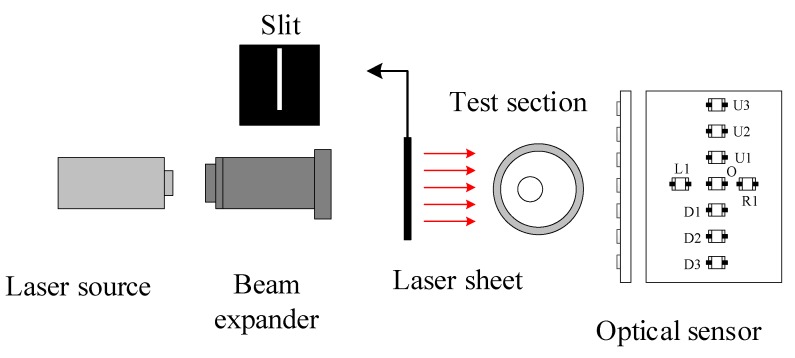
Optical measurement system.

Figure 2 shows the schematic construction of the optical measurement system, which consists of a laser diode, a beam expander, a slit, a test channel, and an optical sensor. The test channel is made of transparent silicate glass in order to allow optical measurement and flow visualization. To achieve a parallel laser beam with small thickness, the beam expander and slit were mounted in front of the laser diode coaxially. The thickness of the slit was set at 1.0 mm to keep the laser beam thin enough and avoid obvious diffraction. Its length was determined according to the diameter of the test channel. After penetrating the test section, the laser beam was scattered by the bubble [13] onto the optical sensor. In the frame of Geometrical Optics, the complex scattering pattern recorded by the optical sensor comes from the reflection and refractions occurring on and through the curved interfaces of the bubble. The laser patterns of different flow patterns were investigated preliminary, and to cover the majority of the laser patterns and maintain the simplest structure possible; the newly developed optical sensor has two crossed arrays. As shown in Figure 2, the vertical array has 7 units, and the horizontal array has 3 units. The height and width of the sensor is 27.7 mm and 14.2 mm, respectively. The sensing unit is PIN photodiode BPW34 (OSRAM), and its sensing area is 3 × 3 mm^2^. The power of the laser diode is 5 mW with a wavelength of 632 nm. The sampling frequency of the optical sensor is 1000 Hz.

## 3. 3D Image Reconstruction Method

**Figure 3:**
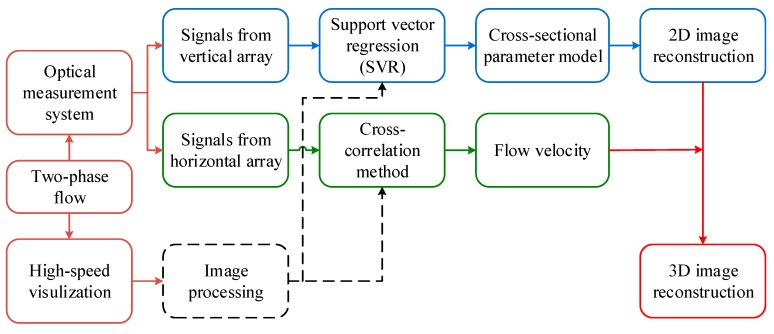
Flowchart of the 3D reconstruction procedure.

Based on the aforementioned optical measurement system, this work proposes a new 3D image reconstruction method for gas-liquid two-phase flow in mini-channels. Figure 3 shows the flowchart of the 3D reconstruction method. First, the signals obtained by the vertical array are used to predict the cross-sectional parameters by a measurement model. The measurement model is developed by Support Vector Regression. Then 2D images, namely the cross-sectional images of the two-phase flow, are reconstructed with the predicted cross-sectional parameters. The flow velocity measurement is implemented by applying the cross-correlation method to the optical signals obtained by the horizontal array, which is further used to determine the spatial interval between the 2D images. Finally, 3D images are reconstructed by piling up the 2D images.

The reference values for model development and velocity measurement are provided by high-speed visualization.

### 3.1. Optical Signal Analysis

**Figure 4:**
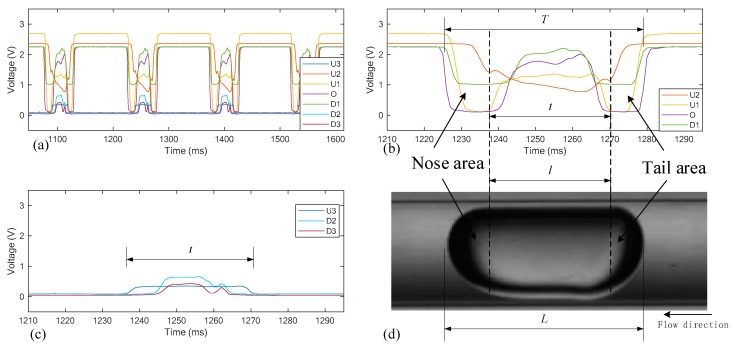
Optical signals of the vertical array: (**a**) optical signals of the vertical array; (**b**) signals of U2, U1, O, and D1; (**c**) signals of unit U3, D2, and D3; (**d**) flipped image of a single slug.

Figure 4a shows a typical group of signals obtained by the vertical array, where O denotes the center unit, U1, U2, and U3 denote the upper ones, D1, D2, and D3 denote the lower ones, *L* denotes the length of the slug, and *l* denotes the length of the slug body. Figure 4b,c show a close-up view of the signals regarding a single slug. The signals of the 7 units display different tendencies, which can be mainly divided into two types: (1) for the central units including O, U1, U2, and D1, the signals have high and steady values during most of the time period, while show a sudden drop and increase at the nose and tail of the slug; (2) for the rest of the units, the signals have relatively low values most of the time and have a relatively smooth increase at the middle part of the slug.

**Figure 5:**
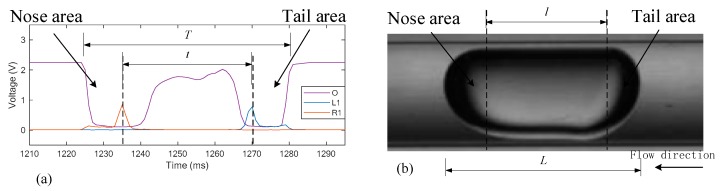
(**a**) Optical signals of the horizontal array; (**b**) flipped image of a single slug.

Figure 5 shows the signals obtained by the horizontal units, i.e., O, L1, and R1, which distribute along the flow direction. Compared with the center unit that is also part of the vertical array, the signals of the rest of the units only have small pulses when the nose and tail pass through the sensing test section. While the rest of the time, the amplitudes of the signals are nearly zero. It is necessary to indicate here that the flow images in Figure 4 and Figure 5 are flipped to match the optical signals. The practical flow direction is from left to right.

**Figure 6:**
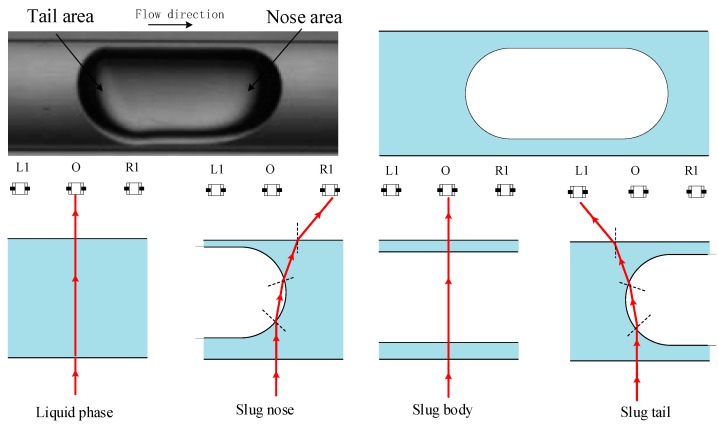
Basic conditions of laser paths related to the interfaces of slug flow.

By referring to the images recorded by the high-speed camera, it is feasible to match the transient optical signals with the cross-section phase boundary and further analyses the effect of the phase boundary on the optical signals qualitatively. Figure 6 shows some basic conditions of the laser path related to the interfaces of the slug flow. According to the figures, it can be seen that when the test section is filled with single-phase water, the signals of the vertical array are stable due to a relatively stable phase interface. When the nose or the tail of the slug reaches the test section, the laser is reflected and diverged due to the curved gas-liquid interface, which decreases the light intensity toward the middle units and results in a sharp decline of the optical signals. However, the diverged laser transmits towards the horizontal array units, which forms the signal pulses. The signals obtained by the vertical array vary according to the cross-section flow structure of the slug flow, which can be further analyzed to determine the parameters of the cross-section. The signals obtained by the horizontal array can reflect the velocity of the flow.

### 3.2. Development of Cross-Sectional Model Based on SVR

**Figure 7:**
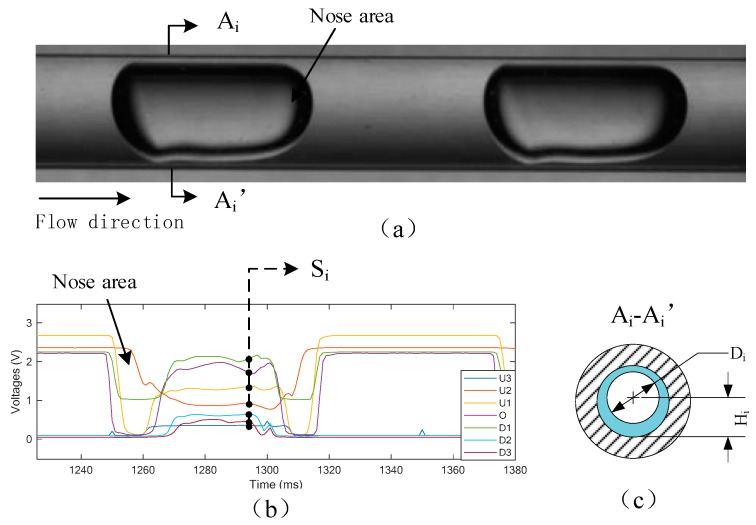
(**a**) Practical image of slug flow. (**b**) Optical signals. (**c**) Cross-sectional parameters.

The cross-sectional model aims to predict the cross-sectional parameters of the slug flow and is developed by Support Vector Regression (SVR). The training data set includes the optical signals obtained by the vertical array and the reference information provided by the high-speed photography. Figure 7a shows an original image of slug flow. Figure 7b,c show the transient optical signals $S_{i}$ of the cross-section A_i_-A_i_’ and its corresponding reference values $D_{i}$ and $H_{i}$. The shape of the gas-liquid interface at the cross-section is mainly determined by the surface tension, which acts to minimize the interfacial area. Therefore, it is reasonable to assume that the shape of the cross-section (slice) of the slug is circular, which is characterized by diameter D and height H. The cross-sectional diameter can reflect the flow structure of the test section, which is important for local void fraction measurement. The cross-sectional height reflects the center position of the gas-liquid interface.

SVR has been widely used to approximate a nonlinear function by mapping input data into a higher dimensional feature space, in which the training data may exhibit linearity [14,15,16]. Given a set of l training points $\left\{\left(x_{1},y_{1}\right),\;\ldots ,\left(x_{l},y_{l}\right)\right\}$, where $x_{i}\in {ℛ}^{n}$ is an input vector of dimensionality n, $y_{i}\in ℛ$ is the target output. The training set is mapped into feature space ℱ by a nonlinear mapping function ϕ(x), which yields the following optimization problem:

(1) $$min_{w,b,\xi ,\xi^{*}}\;\frac{1}{2}\;w^{T}w+C\overset{l}{\underset{i=1}{\sum }}\left(\xi_{i}+\xi_{i}^{*}\right)$$

$$subject\;to\;y_{i}-\left(w^{T}\phi \left(x\right)+b\right)\leq \varepsilon +\xi_{i}$$

$$\left(w^{T}\phi \left(x\right)+b\right)-y_{i}\leq \varepsilon +\xi_{i}^{*}$$

$$\xi_{i},\xi_{i}^{*}\geq 0,\;i=1,2,\ldots ,l$$

where w is a vector variable and b is a threshold variable in the decision function $f\left(x\right)=w^{T}\phi \left(x\right)+b$, $\xi_{i}$, and $\xi_{i}^{*}$ are slack variables that specify the upper and the lower errors in terms of an error tolerance ε, C is a constant that will be discussed later on. In this optimization problem, most data points are expected to be in the ε-tube, otherwise an error $\xi_{i}$ or $\xi_{i}^{*}$ exits [17,18]. By minimizing both the regularization term and the training error term, SVR is capable of avoiding underfitting and overfitting.

To solve the aforementioned optimization problem more easily, its dual problem is introduced by applying Lagrange multipliers $\alpha =\left[\alpha_{1}\;\alpha_{2},\ldots ,\;\alpha_{l}\right]$ and $\alpha^{*}=\left[\alpha_{1}^{*}\;\alpha_{2}^{*},\ldots ,\;\alpha_{l}^{*}\right]$:

(2) $$max_{\alpha ,\alpha^{*}}-\frac{1}{2}\;\overset{l}{\underset{i,j=1}{\sum }}\left(\alpha_{i}-\alpha_{i}^{*}\right)\left(\alpha_{j}-\alpha_{j}^{*}\right)\langle \phi \left(x_{i}\right),\phi \left(x_{j}\right)\rangle -\varepsilon \overset{l}{\underset{i=1}{\sum }}\left(\alpha_{i}+\alpha_{i}^{*}\right)+\overset{l}{\underset{i=1}{\sum }}y_{i}\left(\alpha_{i}-\alpha_{i}^{*}\right)$$

$$subject\;to\;\overset{l}{\underset{i=1}{\sum }}\left(\alpha_{i}-\alpha_{i}^{*}\right)=0\;and\;\alpha_{i},\alpha_{i}^{*}\epsilon \left[0,\;C\right]$$

where, $\langle \phi \left(x_{i}\right),\phi \left(x_{j}\right)\rangle$ is the inner product of $\phi \left(x_{i}\right)$ and $\phi \left(x_{j}\right)$. The computation of the inner product in the feature space may be too complex to calculate. Fortunately, one of the advantages of SVR is that the nonlinear function ϕ(x) need not be used, and the inner product in the feature space can be performed by using a kernel function $K\left(x_{i},x_{j}\right)=\phi \left(x_{i}\right),\phi \left(x_{j}\right)=\phi {\left(x_{i}\right)}^{T}\phi \left(x_{j}\right)$. Any function that satisfies Mercer’s theorem can be used as a kernel function [15]. Radial basis function (RBF) kernel is one of the most widely used kernel functions, which is also used in this work:

(3) $$K\left(x_{i},x_{j}\right)=\exp\left(-\gamma {\left|x_{i}-x_{j}\right|}^{2}\right)$$

The kernel function allows the decision function of the nonlinear problem to be expressed as follows:

(4) $$f\left(x\right)=\overset{l}{\underset{i=1}{\sum }}\left(\alpha_{i}-\alpha_{i}^{*}\right)K\left(x_{i},x\right)+b$$

Cost constant C, error tolerance ε, and the kernel parameter are pre-determined parameters, which are important for the performance of SVR. The parameter ε determines the radius of the ε-tube, outside of which the data are not accepted. ε determines the number of support vectors. C determines the trade-off between the flatness and the amount up to which deviations larger than ε are tolerated. A smaller C may cause errors that are excessively tolerated, resulting in a decision function with poor performance. On the contrary, a greater C may penalize errors too much, which leads to an overfitted function. In this work, we employed the Matlab files coded by Lin from Taiwan University to implement the model development, which is a widely used tool kit [15,19,20].

**Figure 8:**
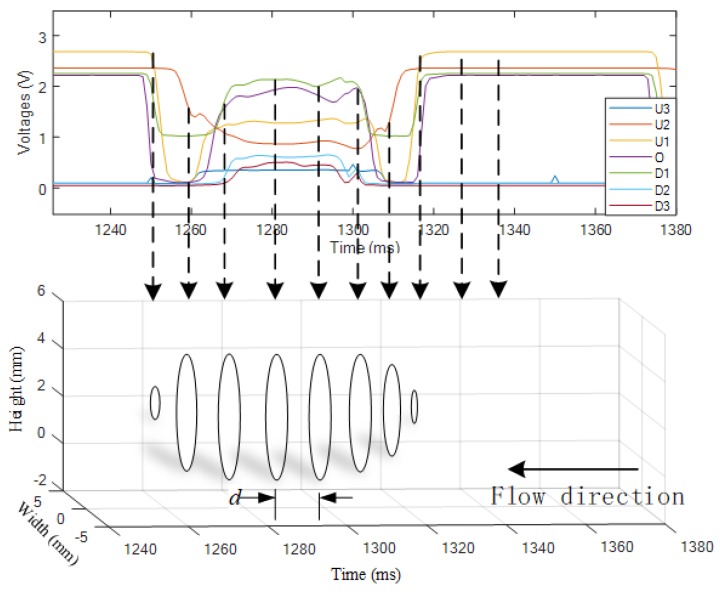
2D reconstruction of the slug flow.

By inputting the measured signals obtained by the vertical array to the SVR model, the cross-sectional diameters and heights are obtained and are further used to reconstruct the 2D images of the slug flow. As shown in Figure 8, one cross-section/slice is accomplished by drawing a circle with the measured cross-sectional parameters. The sequence of the discrete slices builds the outline of the two-phase flow. d denotes the interval between two 2D images that will be determined later on.

### 3.3. Velocity Measurement

The 2D image sequence is arranged by time scale and does not reflect the practical spatial distribution of the slug flow. Hence, flow velocity is needed to adjust the interval between the adjacent 2D images. In conventional cross-correlation methods for velocity measurement, two similar sensors and two light emitters mounted on the upstream and downstream of the flow are necessary to obtain two groups of correlative signals. By calculating the transit time of the two signals, the velocity of the flow can be calculated [21,22]. In this work, a new velocity measurement method is proposed, which only utilizes the horizontal array and one laser source.

The aspect ratio of the slug can be approximately defined as:

(5) $$\beta =\frac{L}{D}=\frac{L}{L-l}$$

where *D* is the maximum diameter of the slug, *L* is the length of the slug, and l is the length of the cylinder body, as shown in Figure 5. By introducing gas velocity *v*, the equation can be rewritten as:

(6) $$\frac{Tv}{D}=\frac{T}{T-t_{0}}$$

where, *T* denotes the time that a slug needs to pass through the test section, and $t_{0}$ denotes the time difference between the two units *L*1 and *R*1. Then, the velocity of the channel can be expressed as:

(7) $$v=\frac{Kd_{i}}{T-t_{0}}$$

where, *K* is a calibration factor that can be obtained by practical experiments, $d_{i}$ is inner diameter of the channel, and $Kd_{i}$ is used to approximate the slug diameter D. The aforementioned derivation is based on the assumption that the slugs share a similar structure.

The cross-correlation method is utilized to calculate the time difference $t_{0}$ between *L*1 and *R*1. Let *S*_1_(*t*) and *S*_2_(*t*) denote the signals from unit *L*1 and *R*1, respectively. The correlation function *R*(*τ*) can be determined by:

(8) $$R\left(\tau \right)=\int_{-\infty }^{+\infty }S_{1}\left(t\right)S_{2}\left(t+\tau \right)dt$$

For discrete signals, the equation can be expressed as:

(9) $$R\left(\tau \right)=\overset{+\infty }{\underset{-\infty }{\sum }}S_{1}\left(n\right)S_{2}\left(n+\tau \right)$$

The time difference of the two signals can be determined by $t_{0}$, which gives R(τ) the maximum value [22].

With the gas velocity v and sampling frequency f, the interval d between the adjacent 2D image can be determined as:

(10) d=v/f

The 3D images of the slug flow are reconstructed by piling up the sequence of 2D images according to the spatial interval d.

### 3.4. Reference Values

**Figure 9:**
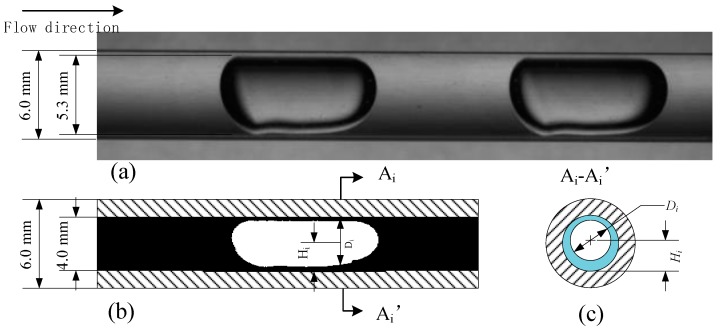
(**a**) Original image of the slug flow; (**b**) binary image; (**c**) cross-section.

The reference values involved in this work, including cross-sectional diameters, heights, and velocities, are provided by high-speed visualization. High-speed visualization is a common technique used to acquire detailed information on the flow structure, especially in academic research. The camera used in this work was MotionXtr N-4 from IDT Redlake. The frequency of the camera was set at 100 fps with a shutter time of 40 μs, the image resolution was reduced to 1016 × 384. It is well known that the recorded images are distorted due to the refraction of the curved channel wall. The optical distortion is more significant in micro/mini channels because of the extremely small dimension of the channel wall. Usually, the images are enlarged compared with the real size of the GLF. As shown in Figure 9a, the actual inner diameter of the channel is 4.0 mm; however, the cross-sectional diameter of the slug in the image obtained by the high-speed camera reaches 5.3 mm, which is 32.5% larger. To minimize the beam steering effect of the curved channel wall, Kawahara suggested an optical correction box filled with a refractive index-matched liquid, which eliminated the enlargement effectively [23]. However, the images are compressed instead. Fu et al. quantitatively investigated the image deformation and calculated that the image would be 20% larger when the refractive indexes of the channel wall and the liquid were 1.52 and 1.21, respectively [6]. This work conducted similar calculations, and the magnification ratio of the image was calculated, which is 31% when the refractive indexes of the channel wall and the liquid are 1.51 and 1.33, respectively. The images obtained by the high-speed camera were corrected by the magnification ratio to eliminate the deformation. It is necessary to indicate that the images only deform in the radial direction. In the axial direction, the images can reflect the flow structure accurately.

After correction, the images are further processed to extract the gas-liquid interface of the interested area. The image processing includes image segmentation, edge detection, and binarization, etc. As shown in Figure 9b, the binary images show the extracted information, where the white pixels represent the gas phase and the black ones represent the liquid phase. The binary images are further analyzed column by column to determine the cross-sectional information. Figure 9c display the cross-sectional information of the cross-section A-A’, where the gas-liquid interface is considered as a circumference with a diameter of *D_i_* and a height of *H_i_*. Gas velocity is calculated by determining the distance that the slug travels in a time period.

## 4. Results and Discussions

### 4.1. Cross-Sectional Parameter Results

**Figure 10:**
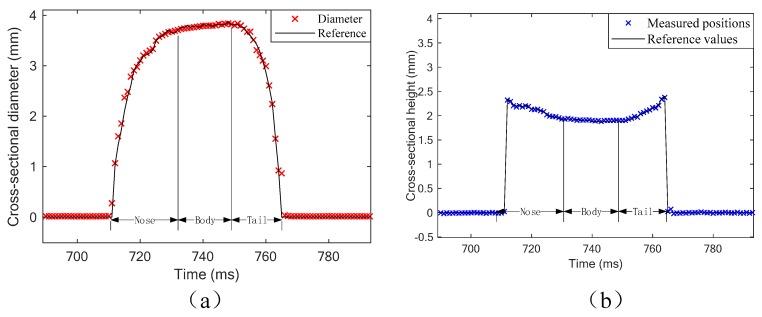
A typical group of experimental results: (**a**) cross-sectional diameter; (**b**) cross-sectional height.

Figure 10 shows a typical group of experimental results of an individual slug, including the cross-sectional diameter and height results. According to the results, it can be seen that the measurement model can predict the cross-sectional interface with satisfactory performance. As shown in Figure 10a, the diameter changes rapidly, and the results have relatively high measurement errors at the nose and tail of the slug. Meanwhile, it can also be seen that the nose is longer than the tail, which can be explained by the different flow mechanism and interactions between the gas and liquid phases at the nose and the tail area. As shown in Figure 10b, the height of the slug body is close to the centerline height of the channel, which is 2.0 mm. However, the tail and nose are over the centerline, which can be explained by buoyancy.

**Figure 11:**
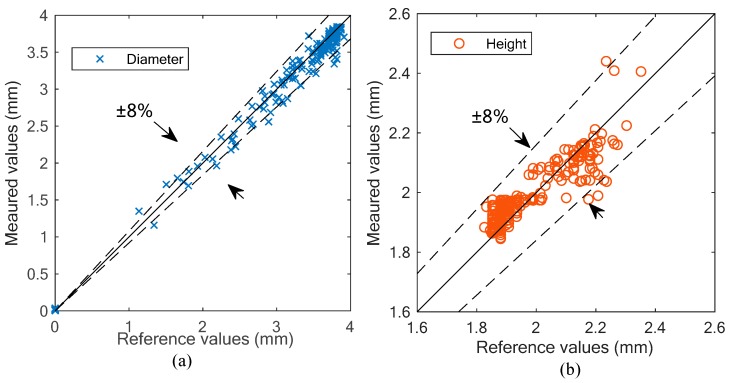
Experimental results: (**a**) cross-sectional diameter; (**b**) cross-sectional height.

Figure 11 shows the relative errors of the diameter and height measurement results. According to the results, it can be seen that both the diameter and height measurements have a maximum relative error of 8%. The results verify the feasibility of the proposed measurement method for cross-sectional parameters. However, there are differences between the two subfigures: in Figure 11a, the results have better accuracy in the area with larger diameters, while in Figure 11b, the area with lower heights have better performance.

In this work, the cross-sectional interface is assumed as circular. This assumption is well satisfied at the body of the slug, where the gas phase occupies most of the cross-section. The body of the slug has a large diameter and low height, and hence the results in this area have good accuracy. However, at the nose and the tail of a slug, the interface is more elliptical rather than circular due to deformation, which leads to relatively high errors.

To implement the measurement of cross-sectional parameters, the reference values obtained by high-speed visualization are needed to form the training data set. Hence, the performance of the measurement is limited by the accuracy of the image processing. Although image deformation has been corrected in this work, more corresponding research is still needed.

### 4.2. Velocity Measurement Results

**Figure 12:**
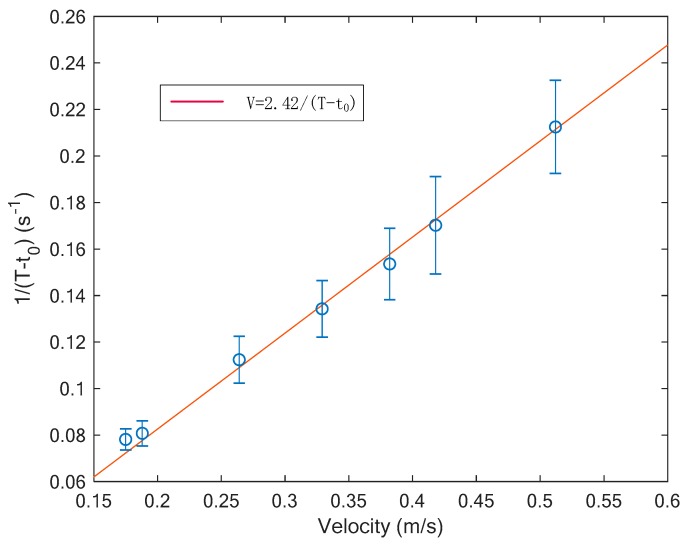
Experimental results of the velocity measurement.

In this work, the velocity of the gas phase is determined by Equation (7). The term K is estimated by the least square method and its value is 0.605. Figure 12 shows the error bar of the velocity measurement. In the range of low velocity, the measurement has small measurement errors. However, in the range of high velocity, the measure errors increase rapidly. The deterioration of the measurement performance can be explained by the sampling frequency of the optical sensor, which was set at 1000 Hz. In the range of low velocity, the sampling frequency is sufficient, and the time T and $t_{0}$ can be calculated with low uncertainty and high accuracy. While in the range of high velocity, a higher sampling frequency is needed, otherwise the time T and $t_{0}$ cannot be accurately measured. Meanwhile, the measurement method relies on the assumption that all the gas slugs share a similar structure. In the range of high velocity, the assumption may not be well satisfied.

The velocity measurement method proposes a new approach to obtain velocity, which only utilizes one laser source and is suitable for restricted space. The experimental results verify the effectiveness of the method in the range of low velocity. For the gas-liquid two-phase flow with high velocity, the performance of this measurement method needs further improvement.

### 4.3. 3D Reconstruction Results

**Figure 13:**
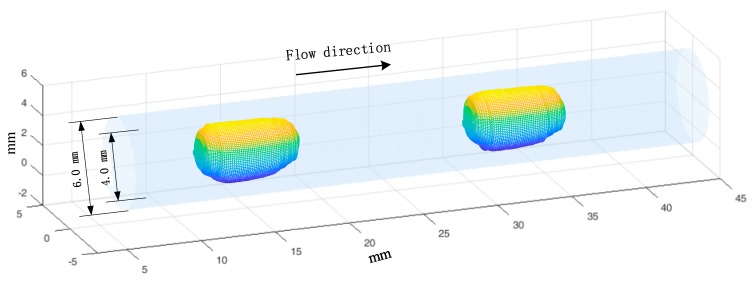
3D reconstruction results of slug flow (velocity 0.15 m/s, void fraction 0.25).

**Figure 14:**
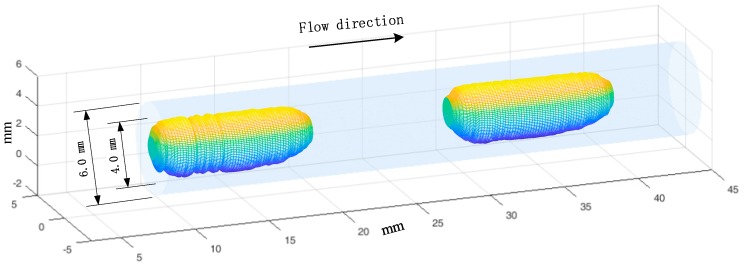
3D reconstruction results of slug flow (velocity 0.42 m/s, void fraction 0.61).

Figure 13 and Figure 14 show the reconstructed 3D images of slug flow in a mini-channel with an inner diameter of 4.0 mm, and the velocities are 0.17 m/s and 0.42 m/s, respectively. The slugs in the images are colored, and the channel wall was added for better viewing. The experimental results verify the effectiveness of the proposed 3D image reconstruction method.

With the introduction of the image correction demonstrated in Section 3.4, the 3D images can display undistorted flow structure of the slug flow in three-dimensional space, which is important for improving the measurement accuracy of other parameters. This method relies on the assumption that the cross-sectional gas-liquid interface is circular. Generally, the surface tension force is dominant compared to other forces, especially for GLF in mini-/micro-channels. Hence, the assumption can be satisfied, and stacking the 2D images can be used to approximate the slug flow.

However, in some uncommon cases, the gas-liquid interface has irregular shapes, which may limit the application of the proposed method. Meanwhile, GLF with high velocities require higher sampling frequency of the optical sensor, which will also affect the 3D reconstruction performance if this is not satisfied.

## 5. Conclusions

The present work proposes a new method for 3D image reconstruction of slug flow in mini-channels based on a new optical sensor. The signals obtained by the sensor are used to determine the gas velocity and cross-sectional interface geometry of slug flow. The 3D images of slug flow are accomplished by piling up 2D images according to a spatial interval. The effectiveness of this method has been verified under the condition of slug flow in the mini-channel with an inner diameter of 4.0 mm.

This method is able to provide detailed information on the flow structure and help to improve the measurement accuracy of some other important parameters such as void fraction, gas-liquid interface, and pressure drop. Compared with other image reconstruction methods, the proposed method has the advantages of low cost, high sampling frequency, and simple structure. The development cost is only 5% of the price of the high-speed camera used in this work, and the required workspace is nearly one-fifth of the camera. Meanwhile, with the use of an integrated photodiode, the optical sensor can be easily multiplied and miniaturized for industrial applications where the working space is restricted.

The 3D image reconstruction is based on the assumption that the cross-sectional interfaces are circular, which is well satisfied in low-velocity slug flow. However, with the increase of velocity, the bubbles will deform, and the assumption becomes inappropriate. The application of this method in other flow patterns, such as bubble flow, annular flow, and stratified flow, needs further investigation. In these flow patterns, the assumption should be reconstructed, and the influence of buoyancy, surface tension, gravity, surface wave, etc., should be reconsidered. In addition, more sensing units, higher sampling frequency, and a thinner laser sheet should also be investigated. Applying the proposed method into the other flow patterns is our next research work.
